# A Comparison of Tissue Handling Forces Between a Novel Suturing Device for Standardised Abdominal Wall Closure and Manual Needle-Driver Suturing

**DOI:** 10.3389/jaws.2025.15377

**Published:** 2025-12-04

**Authors:** G. Börner, E. Lööf, P. Rogmark, M. Edelhamre

**Affiliations:** 1 Department of Surgery, Helsingborg Hospital, Helsingborg, Sweden; 2 Department of Clinical Sciences Lund, Lund University, Lund, Sweden; 3 Department of Surgery, Skåne University Hospital, Malmö, Sweden; 4 Department of Clinical Sciences Malmö, Lund University, Malmö, Sweden

**Keywords:** surgeon skill, suture forces, suturing technique, abdominal wall closure, surgical training

## Abstract

**Introduction:**

Suturing is a fundamental component of surgical procedures, wherein training emphasises the significance of gentle tissue handling. The suturing process involves the pressure exerted by the forceps onto the tissue, as well as the medial traction force applied to stabilise the tissue during the needle bite. This study examined the forces involved in tissue handling during suturing, comparing a novel suturing device for standardised abdominal closure with two sizes of curved suture needles (NDS).

**Methods:**

A model was developed to measure suturing forces. The study introduction comprised both a written letter and an oral explanation. Participants performed 10x3 needle pull-throughs, using a large needle (36 mm, LN) and a small needle (26 mm, SN). Maximum forceps pressure and maximum medial traction forces were recorded. Additionally, needle pull-through time and the area under the curve (AUC) were calculated for both forceps pressure and medial traction pressure.

**Results:**

The study involved 20 specialists, ten scrub nurses, and five surgical trainees. Of these participants, 22 were female, the average glove size was 6.9, and two were left-handed. The use of SutureTOOL resulted in significantly less force exerted with forceps (p < 0.001) when compared to NDS, and a shorter needle pull-through time (p < 0.001). No differences were observed in maximum traction force; however, the medial traction force AUC was lower for SutureTOOL and SN compared to LN (p = 0.025).

**Conclusion:**

The study revealed that SutureTOOL required less forceps pressure and exerted either less or comparable traction force to perform needle pull-throughs, compared to traditional methods. We conclude that this innovative suturing technology did not increase the forces measured in the model. However, the impact on abdominal wall related complications requires further study.

## Introduction

Suturing plays a crucial role in most surgical procedures. In training surgeons, a key emphasis is placed on manipulating tissue as gently as possible to minimise damage and enhance healing [1]. Attention to detail is essential throughout the surgical process, including the placement of the incision, minimal tissue manipulation during surgery, and the application of meticulous closure techniques [2].

Suturing involves the transmission of various forces to the tissue, which can be broadly categorized as follows: First, as the suture needle penetrates the tissue, the forceps apply counter-tension to stabilise it. Second, the interaction between the forceps and the advancing needle generates a horizontal force, leading to medial traction. Third, the tension exerted by the suture thread itself in the approximation of the tissue.

These forces are partly influenced by the friction between the suture and the tissue. Friction can be reduced by shortening the needle, using a smaller diameter needle, polishing the material, and applying a coating [3, 4]. Friction increases with the thickness of the suture thread and when multifilament sutures are used [5]. A fundamental issue with the curved suture needle is the difficulty in achieving a perfect circular suturing pathway while manoeuvring the needle with a needle holder, as the needle becomes buried in the tissue [6]. Surgeons also tend to waver the tip of the curved needle while attempting to reach the intended target [7]. Accuracy in following the intended suture pathway improves, and the force required to advance the needle is reduced when the needle-driver grasps the needle closer to the tip rather than at the non-sharp end [8]. Another method to reduce the force involved in suturing is palm grip suturing. The Frimand needle holder has been shown to reduce surgical stress by 62% compared to conventional needle-driver suturing [9].

In clinical practice, the majority of surgeons often complete laparotomy closure using large-bore sutures (USP 1, 43%–58%, and USP 0, 15%–28%) alongside large needles (LNs) (30–48 mm) [10, 11]. However, this practice does not align with guidelines that recommend the small-bites closure technique, for which the use of a small suture needle is essential [12–14].

When a tissue is manipulated or stabilised, the grasper or forceps exerts a crushing force on it. This crushing force is related to the histologically identified trauma to the tissue [15].

A novel suturing device, SutureTOOL, has been developed to facilitate rapid and standardised abdominal wall closure. The device transports a straight, double-pointed needle between two arms along a track that runs perpendicular to the fascia. This contrasts with the tangential needle track used when suturing with a curved needle, potentially influencing the forces exerted on the fascia during suturing.

The aim of this study was to compare suturing forces using the SutureTOOL with those from manual needle-driver suturing, which was tested using two different sizes of a standard suture needle.

## Methods

**FIGURE 1:**
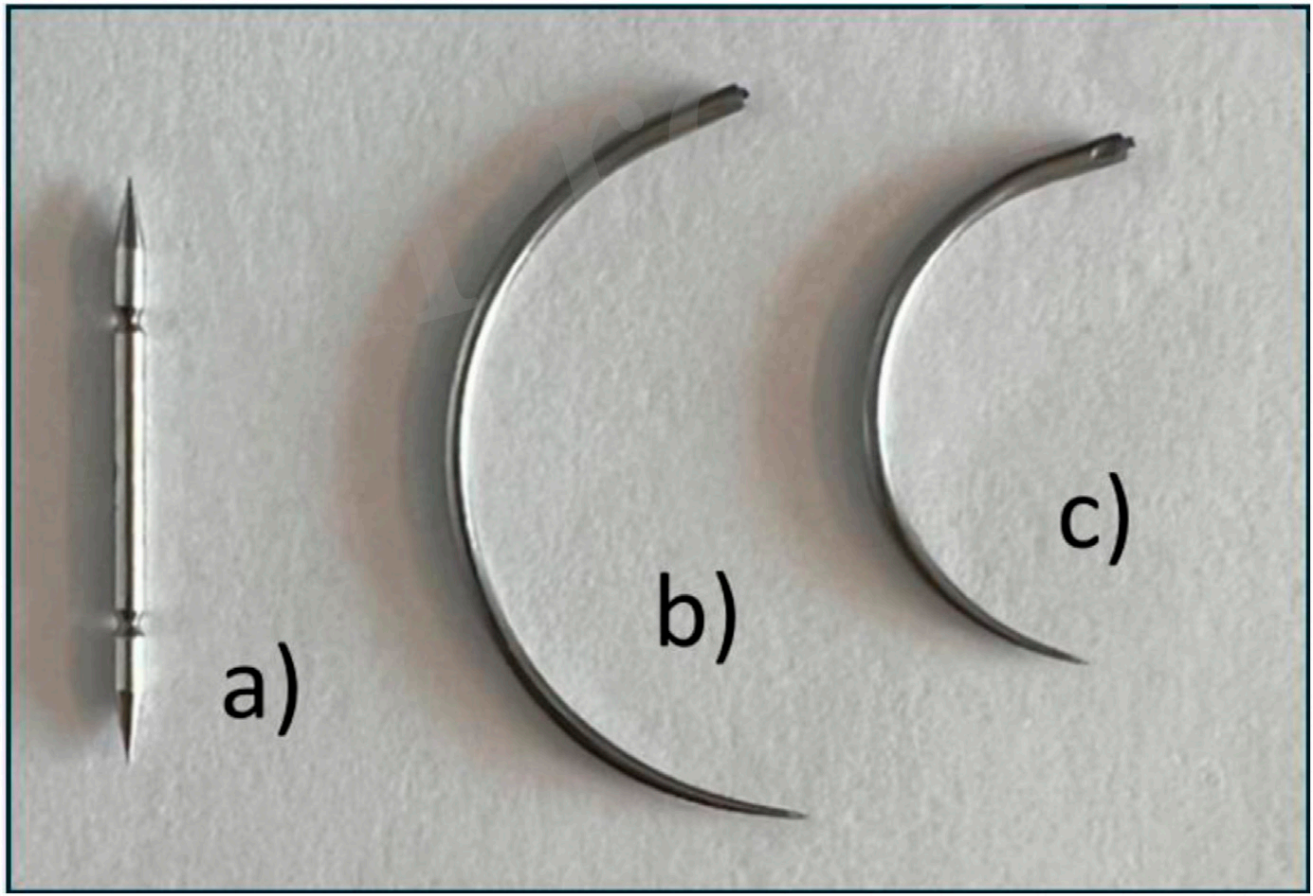
Devices used in this study. **(a)** SutureTOOL double pointed needle, length 20 mm. **(b)** Large semi-circular needle, length 36 mm. **(c)** Small semi-circular needle, length 26 mm. All needles are taper-pointed.

This was an experimental study comparing suturing forces between three different suture needles. Primary endpoint was forceps pressure required to stabilize the tissue. Secondary endpoints included medial traction force and needle pass-through time. The suturing needles assessed included a straight needle, used with the SutureTOOL (Suturion AB, Lund, Sweden), and two different sizes of semi-circular needles (Figure 1). Ethical approval was not necessary for this study.

### SutureTOOL Suturing

**FIGURE 2:**
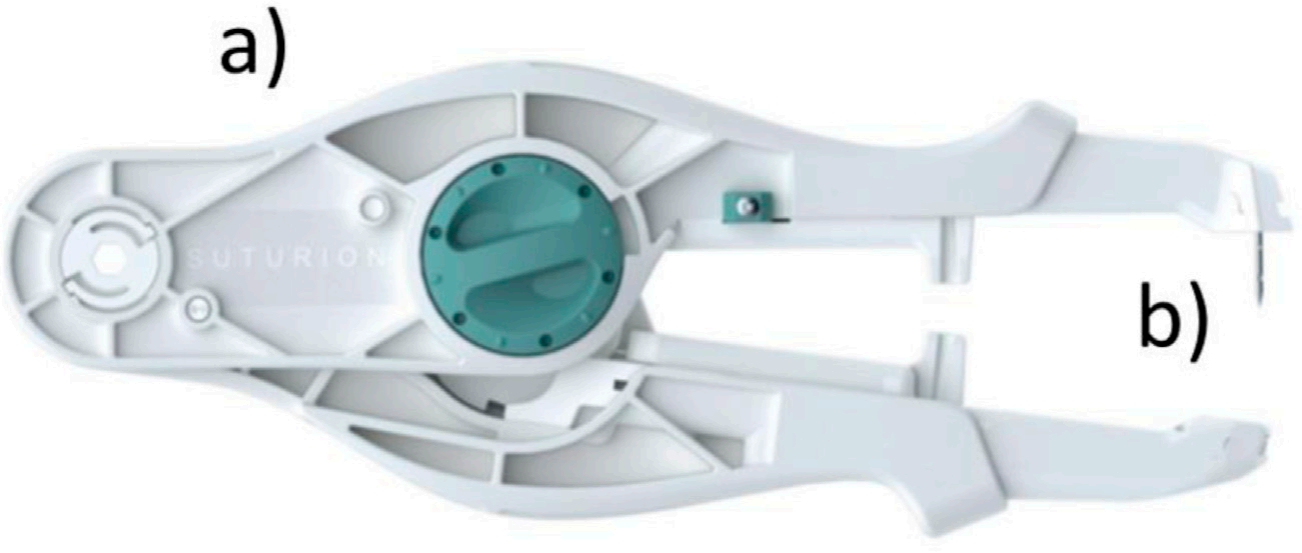
The SutureTOOL. The SutureTOOL handle **(a)** and SutureTOOL double-pointed needle **(b)**. When the device is compressed, the needle is transferred between the jaws and propels the suture thread through the tissue.

SutureTOOL is a handheld suture applicator developed by the first author in collaboration with Lund University (Lund, Sweden). This device is detailed in prior studies [16, 17]. It features a handle and a straight, double-pointed needle with a centrally attached thread (Figure 2). When the device is compressed, the needle transfers between the jaws, enabling the advancement of the suture thread through tissue.

### Needle-Driver Suturing

The manual needle-driver suturing technique (NDS) utilised a standard semi-circular suture needle and a manual needle-driver (Mayo-Hegar 16 cm, Stille AB, Sweden). Two sizes of curved suture needles were employed: a LN (PDS II Suture 2-0, CT-1 needle, Ethicon, Somerville, NJ, USA) and a small needle (SN) (PDS II Suture 2-0, CT-2 needle, Ethicon, Somerville, NJ, USA) (Figures 1b,c). Suture threads were cut close to the needle to enable individual measurement of multiple needle pass-throughs. A new suture needle was used for each participant.

### Theoretical Model for Suturing With Straight and Curved Needles

**FIGURE 3:**
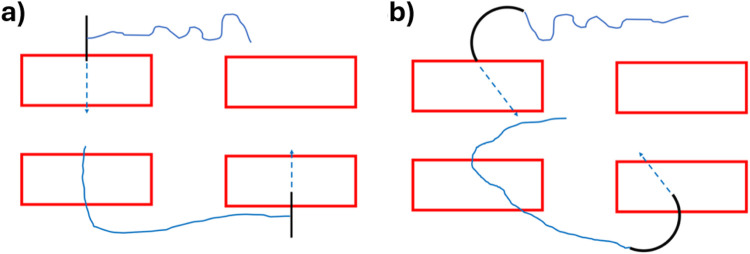
Suture tracks with **(a)** Suture-TOOL suturing with a straight needle **(b)** suturing with a curved needle.

Suturing with the SutureTOOL and a straight needle requires a single stabilising grasp for each tissue bite, with the suture track remaining perpendicular to the tissue (Figure 3a). In contrast, the NDS is controlled by the surgeon’s hand and necessitates two stabilising grasps per bite, alongside repositioning of the needle in the needle-driver. The entry of the suture track is tangential to the tissue surface, possibly resulting in a longer track compared to that of a straight needle (Figure 3b).

### Study Model

The first author developed a technical model designed to measure suturing forces by continuously recording both forceps pressure and medial traction force. A medical student conducted the tests independently of the other authors.

### Forceps Pressure

**FIGURE 4:**
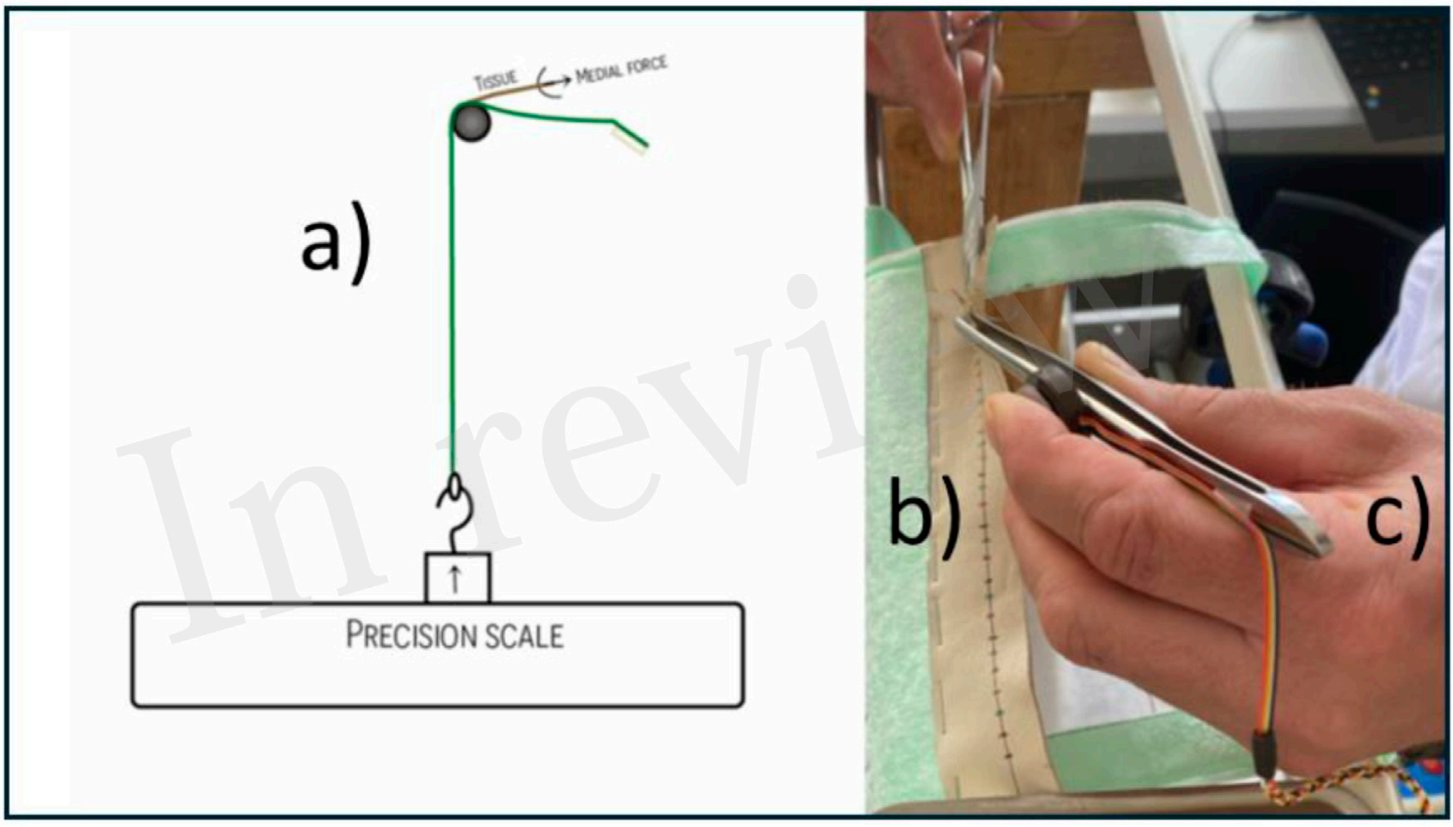
A model to measure suturing forces. **(a)** Suture pad attached to a precision scale. **(b)** Lamb skin suture pad cross-marked for the individual needle pass-throughs. **(c)** Standard forceps with an attached load cell to measure stabilising pressure.

A 4.5 kg load cell (FX29K0-100A-0010-L, TE Connectivity, Berwyn, Pennsylvania, United States) was installed on a 15 cm 3 × 4 claw forceps (Stille, Stille Surgical AB, Solna, Sweden) at the thumb position (Figure 4c). The load cell was connected to a microcomputer (Arduino Nano), and data were transferred and converted into an Excel data file using Serial Plot (Hasan Yavuz Özderya, Hackaday.io). The load cell was calibrated to record ten measurements per second. Before engagement, it transmitted values of approximately 1000 units. The maximum load capacity was 15,000 units, corresponding to 4,536 g.

### Medial Traction Force

The medial traction force was directed from the suturing pad to a precision scale (Model WLC 6/C1/R, Radwag Wagi Elektroniczne, Radom, Poland). Using a precision scale was previously described by Frimand Rönnow [9]. Data were transferred and converted into an Excel data file using Pomair Win (Vetek AB, Väddö, Sweden). The precision scale was configured to record five measurements per second (Figure 4a). The scale provided values in grams, requiring no additional transformation.

### Suturing Pad

A 3 × 25 cm piece of lamb leather served as the suturing pad. Each pad displayed 30 cross-marks in green, blue, and red, spaced at 5 mm intervals along a line located 5 mm from the edge of the pad (Figure 4b). The pad was mounted on a stance specifically developed to measure suturing forces. A new suturing pad was used for each participant.

### Data Analysis

Forceps pressure data and median traction data were adjusted to a baseline of zero and filtered to eliminate signal noise between recorded waves. The duration and maximum amplitude of each individual wave was measured. The AUC for each wave was calculated by summing the amplitudes and multiplying by the measurement interval.

Wave duration, maximum amplitude, and AUC were calculated for the SutureTOOL, LN, and SN. For each participant, a mean value was determined. The data was reported as mean and standard deviation (SD).

The data were analysed using SPSS Statistics for Windows, version 26.001 (SPSS Inc., Chicago, Ill., USA), employing the analysis of variance (ANOVA) procedure. The significance level was set at 5%. For *post hoc* analysis, the Bonferroni adjustment was applied. Data analysis was performed by co-author PR. PR have no financial interest in the investigational device.

### Participants and Instructions

**FIGURE 5:**
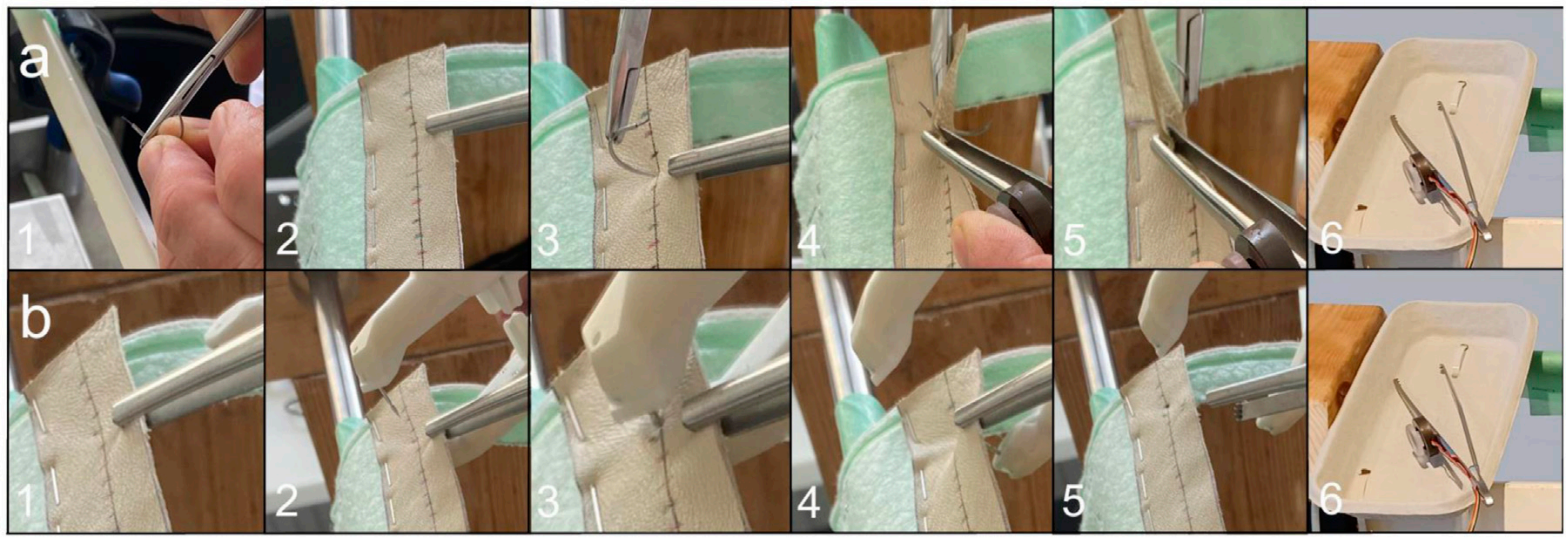
Sequence of a needle pull-through with **(a)** NDS and **(b)** SutureTOOL. **(a)** NDS: 1. Positioning the needle in the needle-driver 2. Grasping the suture pad 3. Penetrating the pad with the curved needle 4. Pushing the needle 5. Pulling the needle through the pad 6. The forceps is placed in the tray. **(b)** SutureTOOL: 1. Grasping the suture pad 2. Positioning the SutureTOOL 3. Compressing the device 4. The straight needle is pulled through the tissue pad by releasing the compression of the SutureTOOL 5. The needle is released from the pad 6. The forceps is placed in the tray.

Specialists in operating specialities, scrub nurses, and surgical trainees were invited to participate and were assigned according to their schedule availability. Participant characteristics collected included age, sex, years in training, subspecialty, glove size, and whether the participant was a nurse or surgeon. The introduction to the study provided both oral and written instructions. Before the test began, participants received brief training on both SutureTOOL and NDS suturing using a separate training model. Individual training time was recorded for SutureTOOL and NDS. Participants performed ten needle pull-throughs with each technique–SutureTOOL and NDS–using LN and SN. Forceps pressure and traction force were recorded for each needle pull-through. The needle pull-through time, as well as maximum force and AUC forces, were calculated. Needle pull-through time was recorded from the first to the last step in the sequences outlined in Figure 5.

## Results

**FIGURE 6:**
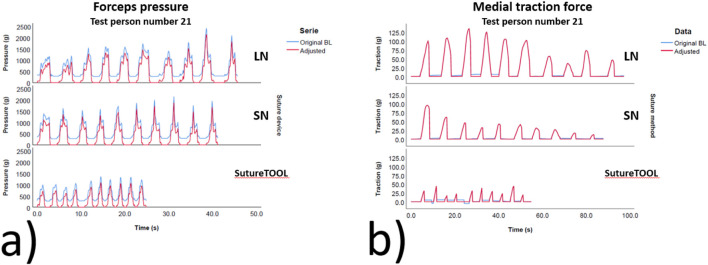
Typical measurements for different suture devices. **(a)** Forceps pressure and **(b)** Medial traction force. Blue waves show the original curve prior to baseline adjustment. Red wave shows waves adjusted to the baseline. g, g s, seconds. LN, large needle (CT-1), SN, small needle (CT-2). BL, baseline.

**FIGURE 7:**
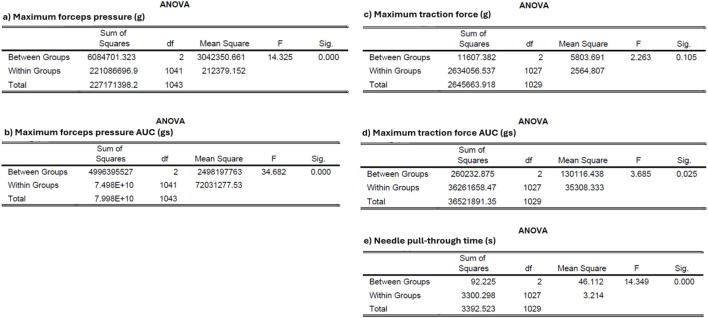
ANOVA table for mean adjusted data **(a)** Maximum forceps pressure **(b)** Maximum forceps pressure AUC **(c)** Maximum traction force **(d)** Maximum traction force AUC **(e)** Needle pull-through time. g, grams. df, degrees of freedom. F, F-statistic. Sig., significance level.

The participants comprised 20 specialists, ten scrub nurses, and five surgical trainees. Of these, 22 were female, with a mean glove size of 6.9, and two participants were left-handed. The training duration was 4 min and 5 s for SutureTOOL and 2 min and 19 s for NDS. SutureTOOL resulted in less pressure force than NDS (p < 0.001) and a shorter needle pull-through time (p < 0.001). Although no differences were found in maximum traction force, the medial traction force AUC was lower for both SutureTOOL and SN compared to LN (p = 0.025). Test outcomes are detailed in Table 1, and typical recordings are presented in Figure 6. Key metrics from ANOVA are depicted in Figure 7.

**TABLE 1:** Test outcomes.

Outcome	SutureTOOL (n = 35)	NDS		*p*
		LN (n = 35)	SN (n = 35)	
Maximum forceps pressure, g	944 (421.9)	1072 (454.3)	1128 (501.6)	p < 0.001
Forceps pressure AUC, gs	1314 (936.8)	1831 (772.9)	1702 (832.2)	p < 0.001
Maximum traction force, g	60 (56.8)	62 (44.8)	54 (49.8)	p = 0.105
Traction force AUC, gs	140 (234.3)	172 (161.3)	139 (160.4)	p = 0.025
Needle-pull through time, s	3.4 (2.15)	4.4 (2.12)	3.9 (1.90)	p < 0.001

Values are mean, standard deviation within brackets. NDS, needle-driver suturing. LN, large needle (CT1). SN, small needle (CT2). g, grams. gs, gram times seconds. AUC, area under curve. P value depicts the difference between groups according to ANOVA, prior to post hoc analysis.

## Discussion

To our knowledge, this is the first study to compare forceps pressure force and medial traction force during suturing with a straight needle and two sizes of NDS needles. The results demonstrated that SutureTOOL required less forceps force compared to both sizes of NDS needles, and it also required lower medial traction force compared to the larger LN needle. These findings are important, as SutureTOOL represents a novel technique for fascia approximation after laparotomy. The forces necessary to stabilise and apply traction to tissue can potentially cause damage. Excessive medial traction force may tear the fascia, increasing the risk of impaired wound healing and incisional hernia formation. The device must not exert greater traction force than the current gold standard, NDS [1]. Although the exact crushing force that fascia can tolerate is unknown, surgeons should adhere to the precautionary principle. Rodrigues et al. investigated the relationship between tractive forces and tissue damage in different porcine abdominal organs [1]. Authors found that fascia and aorta had the highest tolerance, while uterus and fallopian tube had the lowest. Fascia was damaged at a median tractive force of roughly 11.5 N. The maximum mean traction force in this study was 62 g and the highest recorded value was 440 g, which corresponds to 0.6 N and 4.3 N respectively. Assuming the findings are comparable, the forces recorded in the study was less than at risk of rupturing the fascia.

The medial traction force was lower for SutureTOOL and SN compared to LN. LNs are commonly used for laparotomy closure following open surgery, despite the recommendation for smaller needles to facilitate the small-bites closure technique [10, 11, 14]. A major disadvantage of LNs is their use of unnecessarily large-bore suture threads, which result in higher tissue friction [5]. Several clinical trials have demonstrated that a suture bore of USP 2/0 is sufficient for the small-bites closure technique [18–20]. Small needles offer the advantage of taking smaller tissue bites and encourage the surgeon to limit the bite to the midline aponeurosis exclusively. The study indicates that small needles reduce the amount of force imposed on tissue, which can be beneficial for wound healing.

Incision closure time has previously been shown to be shorter with SutureTOOL, with 95%–100% adherence to the small-bites closure technique demonstrated in animal tissue models, cadaver models, and clinical trials [16, 17, 21]. In this study, the pull-through time with SutureTOOL was shorter compared to LN and SN. Laparotomy closure using the small-bites technique after open surgery can take 18–30 min [18, 22]. Reducing small-bite closure time is crucial for implementing this technique, as some surgeons consider the extra time required an obstacle [23]. A reduction in laparotomy closure time also provides an opportunity to reallocate limited surgical resources to more patients.

Many surgical tasks rely heavily on individual proficiency. Seki introduced the term *surgeotechnology* to underscore the importance of teaching and assessing basic surgical skills, such as suturing and knot tying, which can significantly impact patient outcomes [7]. Conway demonstrated considerable variation among surgeons when estimating the distance for suture placement [24]. In a study by von Trotha regarding knot tying, the final tension varied from 0.19 to 10 N among 118 surgeons, highlighting the substantial differences in this fundamental skill [25].

Several limitations of the study need to be addressed. The lamb patches were cut from a single sample of leather, why the individual patches were considered comparable in strength. Lamb skin is probably tougher to penetrate compared to human fascia due to it being tanned and lacks lubrication and an exaggeration of the measured forces would be expected. The clinical relevance of the study is not predictable by the findings. The aim was to assess if the straight needle, as being part of a novel fascia closure method, imposed more forces compared to the standard curved needles. The study found comparable forces among the studied needles in the model and the potential difference in tissue damage and healing needs to be addressed in future clinical studies.

Another limitation of the study is the limited training. The scrub nurses have less experience in suturing and the overall results would probably have benefited from performing the study after formal training and proficiency examination.

The SutureTOOL propels the straight needle between its arms perpendicularly, unlike the NDS, where the needle is passed through tissue tangentially. Furthermore, the surgeon does not need to manipulate the needle to reach the target, as the SutureTOOL includes a guide for placing small bites with high accuracy [21]. According to Seki, handling a curved needle in tissue involves wavering the needle tip, which may potentially harm the tissue [7]. Although the clinical relevance of this wavering is unknown, it would be worthwhile to compare the passage accuracy through tissue of the SutureTOOL and NDS in a future study.

Surgical procedures are often lengthy and strenuous. The risk of complications rises with extended operating times, and there is a correlation with surgeon fatigue [26, 27]. The SutureTOOL requires fewer steps to place the suture line, and adherence to small-bite techniques is high. The next steps involve studying adherence to the small-bites closure technique and comparing surgical site occurrences between the SutureTOOL and NDS in settings involving patients undergoing long operations, such as debulking or transplantation surgery.

## Conclusion

The study demonstrated that SutureTOOL required less forceps pressure and exerted equal or reduced traction force for needle pull-throughs compared to traditional suturing methods. We conclude that this innovative suturing technology did not increase the forces measured in the model. However, the impact on abdominal wall related complications requires further study.

## Data Availability

The raw data supporting the conclusions of this article will be made available by the authors, without undue reservation.
